# Bone demineralization in a cohort of Egyptian pediatric liver transplant recipients: Single center pilot study

**DOI:** 10.1097/MD.0000000000031156

**Published:** 2022-11-11

**Authors:** Magd A. Kotb, Lubna A. Fawaz, Rania A. Zeitoun, Yomna M. Shaalan, Nazira Aly, Hesham Abd El Kader, Gamal El Tagy, Haytham Esmat, Alaa F. Hamza, Hend Abd El Baky

**Affiliations:** a Department of Pediatrics, Cairo University, Cairo, Egypt; b Department of Radiology, Cairo University, Cairo, Egypt; c Department of Pediatric Surgery, Ain Shams University, Cairo, Egypt; d Department of Pediatric Surgery, Cairo University, Cairo, Egypt.

**Keywords:** bone health, DEXA scan, orthotopic liver transplant

## Abstract

Liver transplantation (LT) is the definitive treatment of end-stage liver disease. The long-term survival following LT spurred more interest in improving the quality of life of patients. This was a cohort study that included 23 pediatric liver transplant recipients who underwent LT due to hereditary or metabolic liver diseases. Bone health assessment was performed at their last follow up clinically (anthropometric measures), biochemically and radiologically (Dual Energy X-ray Absorptiometry [DEXA] scans). Poor bone health was defined as z-score <−1. Mean age at LT was 5.77 years (standard deviation [SD] 3.64) and 43% were males. Biliary atresia was the most common cause of end stage liver disease (35%). Mean age at follow up was 14 years (SD 5.48) and mean follow up was 8 years (SD 4.12 years). Eleven patients (48%) had poor bone health (osteopenia 22% and osteoporosis 26%). On univariate analysis, being on steroids at last follow up (odds ratio [OR] 13.2, 95% confidence interval [CI] 1.23–140.67, *P* = .03), weight at last follow up (OR 0.45, 95% CI 0.20–0.99, *P* = .04), platelets at last follow up (OR 0.98, 95% CI 0.96–s0.99, *P* = .02), hemoglobin at last follow up (OR 0.33, 95% CI 0.12–0.89, *P* = .03) were significantly associated with poor bone health. None of the variables were significant on multivariate analysis. At most recent follow up, 48% of patients demonstrated poor bone health by DEXA scans. More studies are required to evaluate predictors of poor bone health after LT in children.

## 1. Introduction

Twelve percent of orthotopic liver transplants (OLT) occur in pediatric patients.^[1]^ Survival rates following OLT witnessed paramount improvement (94% patient survival and 84% graft survival at 5 years) owing to advances in surgical techniques and immunosuppression.^[2,3]^ Therefore, evaluation of outcomes of pediatric OLT and prevalence of long-term complications are critical to understand the long-term needs of this unique patient population, including the effects on growth and bone health.

Before OLT, bone health is negatively affected by the preexistent liver disease.^[4]^. The combination of malnutrition, malabsorption, impaired hepatic functions, poor mobility and hypogonadism can cause growth failure and bone complications. After transplant, liver functions are restored, but may still be counteracted by other factors such as abnormal thyroid profile, diabetes mellitus, pubertal delay,^[5–9]^ poor postoperative ambulation,^[4]^ and prolonged large doses immunosuppression.^[10,11]^

Hepatic osteodystrophy refers to the metabolic bone derangement associated with liver disease. In adults, it commonly involves osteoporosis, fractures, and osteomalacia. In growing children, it affects existing bone material in addition to growth plates resulting in low bone mass, fractures, rickets, spine abnormalities and growth failure.

Most of the data about bone health in children has been extrapolated from the adult literature, which may not be clinically appropriate.^[12]^ For example, bone mineral density (BMD) measured by Dual Energy X-ray Absorptiometry (DEXA) scan in adults is commonly reported as a T-score, representing the deviation from the mean peak young adult value for BMD.^[13]^ Physiologic growth in children involves continuous building of bone mass. Therefore, comparing their BMD to adults seems inaccurate. In children, z-scores are typically used to report BMD values compared with the age- and sex-matched peers.

Many factors can contribute to bone-related complications after OLT, but still poorly understood. The aim of the current study was to describe the effect of OLT on bone health utilizing DEXA scans and to identify patient, disease- and treatment-related factors that may associate with poor bone health.

## 2. Methods

This was a single institution, cross-sectional descriptive study that included children with end stage liver disease who underwent OLT from living donors in the Pediatric Liver Transplant Unit at Wadi El Neel Hospital, Egypt. The study was approved by The Pediatric Department Committee for Post- Graduate Studies and Research, and by Post-Graduate Studies and Research Administration, Faculty of Medicine, Cairo University, Egypt. The study complies with Declaration of Helsinki - Ethical Principles for Medical Research Involving Human Subjects.^[14]^ Informed consent was obtained from all participants and/or their legal guardians.

A total of 23 pediatric liver transplant recipients who underwent liver transplant due to hereditary liver diseases (e.g., extrahepatic biliary atresia) or metabolic diseases (e.g., Tyrosinemia) were included in our study. All were younger than 18 years at time of transplant with a minimum follow up of 6 months post-transplant. Recipients who were older than 18 years at time of transplant or who followed up for less than 6 months after transplant were excluded from the study.

Patient demographics and disease characteristics were obtained by chart review including pre-operative variables (gender, age at transplantation, blood group, Pediatric End Stage Liver Disease score, etiology of end stage liver disease, donor relation and gender), operative and post-operative variables (post-operative intensive care unit and hospital stay, complications and immunosuppressive medications received). Pediatric end stage liver disease score was calculated from serum albumin, bilirubin, International normalized ration, age at listing and failure to thrive based on weight, height and gender.

Bone health was assessed clinically. Anthropometric measurements were expressed as standard deviation (SD) score (z-score) which is defined as deviation of height or weight, in SD units, from the mean height or weight for age and sex. SD or z-scores were calculated using a computer system (Growth Vision 2.0) at the Endocrinology Clinic, Cairo University.^[15]^ Body mass index (BMI) was calculated as (weight in kilograms/height in meters^2^) and expressed in kg/m^2^. SD (z-scores) were calculated using the same program as weight and height.

Biochemical assessment of bone health was performed by obtaining serum calcium, phosphorus, magnesium, parathormone, vitamin D levels, sodium, potassium, chloride as well as calculating the anion gap in addition to routine follow up labs such as complete blood count and liver enzymes.

Radiological assessment of bone health included DEXA scan to assess the bone mineral content. BMD measurements were performed at the lumbar spine. The value of BMD was reported as z-score (SDs from the mean of a healthy age- and sex-matched reference population). Values calculated were compared to normal individuals of the same age and sex among Egyptian children at the Radiology Department, Cairo University. Osteopenia was defined as a z-score between 1 and 2 below the mean for age and sex. Osteoporosis was defined as z-score < −2.^[16]^ History of fractures was also collected from the database and patient’s interview.

The data were analyzed using the Statistical Package for the Social Sciences (SPSS version 21). Data were presented using descriptive statistics (mean, SD, median and frequency where appropriate). Height, weight and BMI were transformed into SD scores or z-scores using Growth Vision 2.0 software package. Patients with normal bone health were compared to those with osteopenia and osteoporosis using Mann–Whitney, *t* test, Pearson Chi square test and Fisher’s Exact test where appropriate. Univariate and multivariate logistic regression analyses were performed to model variables associated with poor bone health. Two-sided *P* values ≤ .05 was considered significant.

## 3. Results

A total of 23 pediatric liver transplant recipients were included in our study. Mean age at OLT was 5.77 years (SD 3.64) and 43% were males. Biliary atresia was the most common cause of end stage liver disease (35%). At last follow up, mean age was 14 years (SD 5.48) and mean follow up was 8 years (SD 4.12 years). Mean height standard deviation score (SDS) was −0.60 cm (±1.68) and mean weight SDS was 0.17 kgs (±1.8) (Table 1). The majority of patients (n = 18, 75%) were on multiple immunosuppressive medications with tacrolimus being the most commonly received (n = 21, 88%) (Table S1, http://links.lww.com/MD/H615). History of fractures was obtained together with laboratory and radiological assessment of bone by DEXA scan. Only 3 patients (13%) had history of a fracture, 2 (8.6%) in the forearm and 1 (4.3%) in the lower limb. Laboratory assessment included vitamin D level which was deficient in 18 (86%) of patients with the mean value 12.79 (±10.63). Mean anion gap was 9.6 (±4.22) (Table S2, http://links.lww.com/MD/H616).

**Table 1:** Clinical data of patients presented to the annual follow up.

N of patients	23
Age at last follow up, mean (SD) (yrs)	13.97 (±5.48)
Duration of follow up, mean (SD) (yrs)	8.19 (±4.21)
Height SDS, mean (SD) (cm)	−0.60 (±1.68)
Weight SDS, mean (SD) (kg)	0.17 (±1.8)
BMI SDS, mean (SD) (kg/m^2^)	0.02 (±1.8)

BMI = body mass index, SD = standard deviation, SDS = standard deviation score.

DEXA scan results showed a mean z-score of −1.3 (±1.5); 12 patients (52.2%) showed a normal DEXA scan, 5 patients (21.7%) had osteopenia and 6 patients (26.1%) had osteoporosis (Tables S3, http://links.lww.com/MD/H617 and S4, http://links.lww.com/MD/H618). Patients were divided into 2 groups; those with normal DEXA scan results and those with abnormal DEXA scan results (osteopenia and osteoporosis). Pre-transplant variables were compared among the 2 groups. Patients with normal DEXA scan had a median pre-operative weight of −0.9 compared to −1.8 in patients with abnormal DEXA (*P* = .04). There was no significant difference among the remaining variables (Table 2).

**Table 2:** Pre-transplant variables were compared among patients with normal and abnormal DEXA scan.

Preoperative parameters	Normal DEXA scan N = 12 (52.2%)	Abnormal DEXA scan N = 11 (47.8%)	*P* value
Age at transplantation, median (%) (yr)			.68
<5 yrs	6 (50)	7 (63.6)	
>5 yrs	6 (50)	4 (36.4)	
Gender			.1
Males, n (%)	3 (25)	7 (63.6)	
Females, n (%)	9 (75)	4 (36.4)	
Height SDS, median (range) (cm)	−1.75 (−3.9 to −1)	−1.8 (−4.6 to −0.4)	.64
Weight SDS, median (range) (kg)	−0.9 (−3 to 1.1)	−1.8 (−3.4 to −0.6)	.04
Body mass index SDS median (range) (kg/m^2^)	0.9 (−1.1 to 2.5)	−0.08 (−2.9 to 1.5)	.11
Blood group, n (%)			.86
A	6 (54.5)	5 (45.5)	
B	3 (27.3)	3 (27.3)	
O	2 (18.2)	3 (27.3)	
AB	0	0	
Serum total bilirubin, median (range)	5.9 (0.2–38.5)	7.5 (0.2–24.7)	.8
Serum direct bilirubin, median (range)	1.7 (0.1–24)	4.7 (0–17.3)	.66
Serum albumin, median (range)	3.9 (2.6–4.7)	3.2 (1.8–4.7)	.17
Serum creatinine, median (range)	0.4 (0.2–6.7)	0.4 (0.2–4.6)	.97
Serum ALT folds, median (range)	1.15 (0.47–2.82)	1 (1–3.25)	.67
PT, median (range)	16.25 (12–23)	15.9 (12–34.6)	.82
INR, median (range)	1.4 (1–2.1)	1.29 (1– 3.6)	.35
PELD score	9.5 (0–21)	10 (0–27)	.47
PELD score, n (%)			.54
<18	9	8	
18–24	3	2	
>24	0	1	
Cause of liver disease, n (%)			.82
Biliary atresia	4 (33.3)	4 (36.4)	
Autoimmune hepatitis	1 (8.3)	0	
Familial hypercholesterolemia	2 (16.7)	1 (9.1)	
Congenital hepatic fibrosis	0	1 (9.1)	
PFIC	0	3 (27.3)	
Unresolved neonatal hepatitis	1 (8.3)	0	
Wilson’s disease	1 (8.3)	0	
Galactosemia	0	1 (9.1)	
Primary hyperoxaluria	1 (8.3)	0	
Tyrosinemia	1 (8.3)	0	
Criggler Najjar	1 (8.3)	1 (9.1)	
Donor, related, n (%)	9 (75)	7 (63.6)	.66
Donor gender,			.22
Males, n (%)	4 (33.3)	7 (63.6)	
Females, n (%)	8 (66.7)	4 (36.4)	

ALT = alanine transaminase, DEXA = dual energy X-ray absorptiometry, INR = international normalized ration, PELD = pediatric end stage liver disease, PFIC = progressive familial intrahepatic cholestasis, PT = prothrombin time.

Operative and post-operative variables were also compared. Among those with normal DEXA scan, only 1 patient restarted steroids after stopping it, while 5 patients did among the abnormal group with a trend towards significance (*P* = .06) (Table 3). Patients with abnormal DEXA scan had worse median weight SDS (−0.6 in the abnormal group compared to 0.5 in the normal group, *P* value = .03), BMI SDS (−0.4 in the abnormal group compared to 1.1 in the normal group, *P* value = .03), and were receiving steroids more frequently (6 in the abnormal group compared to 1 in the normal group, *P* = .02). They had lower hemoglobin levels (11.8 vs 13.1, *P* = .01) and lower platelet count (167 vs 250, *P* = .01) (Table 4). Laboratory assessment for bone health was also compared. Serum Chloride was higher among the abnormal bone health group (104 vs 102, *P* = .03). There was no significant difference in the number of fractures and other lab values (Table S5, http://links.lww.com/MD/H619).

**Table 3:** Operative and postoperative variables among patients with normal and abnormal DEXA scan.

Operative and postoperative outcomes	Normal DEXA scan	Abnormal DEXA scan	*P* value
ICU stay, median (range) (d)	10.5 (4–21)	10 (6–32)	.87
Room stay, mean (SD) (d)	13 (1–35)	17 (3–23)	.62
Total duration in hospital median (range) (d)	21.5 (12–54)	27 (14–40)	.26
Complications, n (%)	8 (66.7)	9 (81.8)	.64
Acute rejection	2 (16.7)	4 (36.4)	.37
Chronic rejection	4 (33.3)	6 (54.5)	.41
Biliary complications	1 (8.3)	3 (27.3)	.31
Vascular complications	2 (16.7)	3 (27.3)	.64
Immunosuppression regimen, n (%)			.47
Steroids/Tacrolimus/MMF	12 (100)	10 (90.9)	
Tacrolimus	0	1 (9.1)	
Patients received steroids	12 (100)	10 (90.9)	.47
Patients stopped steroids	11 (91.7)	10 (90.9)	.94
Cause of discontinuation			.94
Schedule	11 (100)	10 (100)	
Patients regained steroids after stopping it	1 (8.3)	5 (50)	.06
Patients stopped steroids again	1 (100)	0	.32
Total duration of steroids received median (range) (mo)	6 (6–132)	6 (0–120)	
<6	10	6	.31
>6	2	5	.13

DEXA = dual energy X-ray absorptiometry, ICU = intensive care unit, MMF = mycophenolate mofetil, SD = standard deviation.

**Table 4:** Variables at last follow up among patients with normal and abnormal DEXA scan.

Variables at last follow up	Normal DEXA	Abnormal DEXA scan	*P* value
Age at last follow up, median (range) (yrs)	14.02 (6.33–24)	12.6 (5.53–21.64)	.6
Duration of follow up, median (range) (yrs)	7.02 (1–16)	8.5 (1–13)	.95
Height SDS, median (range) (cm)	−0.7 (−2.2 to 1.3)	−0.8 (−5.5 to 3.3)	.26
Weight SDS, median (range) (kg)	0.5 (−1 to 5.5)	−0.6 (−2.9 to 1.2)	.03
BMI SDS, median (range) (kg/m^2^)	1.1 (−1.4 to 2.2)	−0.4 (−5.7 to 2.1)	.03
Serum total bilirubin, median (range)	0.7 (0.24–17)	0.7 (0.3–4.5)	.97
Serum direct bilirubin, median (range)	0.2 (0.08–16)	0.3 (0.09–1.6)	.36
Serum albumin, median (range)	4.15 (4–4)	4.1 (2–5)	.55
Serum creatinine, median (range)	0.51 (0.3–1)	0.53 (0.5–17)	.42
Serum Urea, median (range)	24.5 (3–43)	19 (1–39)	.4
Creatinine clearance, median (range)	98.6 (46–239)	68.9 (0–121)	.15
Serum AST folds, median (range)	1 (1–9)	1 (1–5)	.38
Serum ALT folds, median (range)	1 (1–6)	1 (1–5)	.7
Serum GGT folds, median (range)	1 (1–15)	1.43 (1–5)	.07
Serum PT, median (range)	13.4 (10.1–15.7)	14.2 (10.3–16.8)	.27
Serum INR, median (range)	1.07 (0.9–1.44)	1.1 (0.8–1.39)	.59
Serum Na, median (range)	138 (131–146)	137.5 (126–143)	.71
Serum K, median (range)	4.2 (3.7–4.6)	4.25 (3.7–5.5)	.28
Serum HCO_3_, median (range)	23.1 (21.2–26.60)	23.7 (18.8–29.5)	.87
Serum glucose, median (range)	97.5 (88–109)	86 (75–122)	.29
Hb, median (range)	13.1 (10.8–14.1)	11.8 (9.5–13.2)	.01
Hct, median (range)	37.1 (33–43)	35 (28–46)	.21
Platelets, median (range)	250 (133–365)	167 (28–327)	.01
WBCS, median (range)	7.1 (4–9.7)	6.5 (2.8–9.5)	.46
Immunosuppression, n (%)			.31
Single	4 (33.3)	1 (9.1)	
Multiple	8 (66.7)	9 (81.8)	
None	0	1 (9.1)	
Types of immunosuppression, n (%)			
Steroids	1 (8.3)	6 (54.5)	.02
Imuran	2 (16.7)	5 (45.5)	.19
Prograph	12 (100)	9 (81.8)	.21
Cellcept	6 (50)	5 (45.5)	1
Everolimus	0	1 (9.1)	.47

ALT = alanine transaminase, AST = aspartate aminotransferase, DEXA = dual energy X-ray absorptiometry, GGT = gamma glutamyl transferase, INR = international normalized ration, PT = prothrombin time, SDS = standard deviation score, WBCs = white blood cells.

On univariable analysis, pre-operative weight SDS (odds ratio [OR] 0.35; confidence interval [CI] 0.12–1.02; *P* = .05), Weight SDS at last follow up (OR 0.45; CI 0.2–0.99; *P* = .04), Hb at last follow up (OR 0.33; CI 0.12–0.89; *P* = .03), Platelets at last follow up (OR 0.98; CI 0.96–0.99; *P* = .02) and steroids (OR 13.2; CI 1.23–140.6; *P* = .03) were significantly associated with low BMD (Table S6, http://links.lww.com/MD/H620). On multivariable analysis, none of the variables was significant, with platelets count at last follow up showed a trend towards statistical significance (OR 0.95; CI 0.90–1.00; *P* = .09) (Table S7, http://links.lww.com/MD/H621).

## 4. Discussion

Assessment of bone health in pediatrics undergoing OLT remains a challenge. First, there is lack of standardized definition for what constitutes normal bone health in children, especially those 2 years or younger.^[17]^ Second, most of the literature about bone health following OLT in the pediatric population is derived from adult literature. Third, there is lack of prospective trials and most current data is derived from retrospective studies with its known limitations.

Hepatic osteodystrophy in the pediatric population comprises mainly fractures, low bone mass, vitamin D deficiency rickets, avascular necrosis and scoliosis. Fractures before OLT occurs in up to 28% of children (compared to none among the 23 patients in our study).^[18,19]^ After OLT, up to 38% of children (compared to 13% in the current study) have history of fractures.^[18–21]^ In agreement with our findings, most fractures among the pediatric population were reported to be non-vertebral.^[4,21–23]^ Data from the adult literature showed up to 35% fractures before OLT and 65% after OLT, with most fractures being vertebral and asymptomatic. Interestingly, recent studies using spine X-rays and vertebral morphometry suggested similar pattern of vertebral fractures in pediatrics.^[4,21]^ In the current study, bone health evaluation was performed using history taking, laboratory assessment and DEXA scans and did not include spine X-rays or vertebral morphometry.

BMD measured by DEXA scan in adults is commonly reported as a T-score. The World Health Organization defines osteoporosis as a T-score < 2.5.^[13]^ The difference in definitions of abnormal BMD in children may contribute to the variation in prevalence of low BMD after OLT. In the current study we utilized a more stringent definition of low BMD. Osteopenia was defined as a z-score between 1 and 2 below the mean for age and sex. Osteoporosis was defined as z-score < −2. The term low BMD in the current study included both osteopenia and osteoporosis.^[16]^ Other studies utilized more flexible definitions relying on both clinical and densitometric parameters. The International Society for Clinical Densitometry 2007 Position Development Conference mandated the presence of both a clinically significant fracture history (long bone fracture of the lower extremities, vertebral compression fracture, or 2 or more long-bone fractures of the upper extremities) and low BMD (z-score ≤ −2) for the diagnosis of osteoporosis.^[24]^

In adults, low BMD is observed in 16% to 79% of patients before OLT.^[25,26]^ Rapid bone loss occurs in the first 3 to 6 months after OLT, followed by slow and sometimes partial recovery of BMD.^[18,27–29]^ While BMD changes in children are generally similar to adults, there is a higher potential for spontaneous recovery.^[25,30–32]^ Nevertheless, a longer time and/or incomplete recovery (BMD z-score < −2) occur in 0% to 15% of long-term survivors after OLT.^[4,25]^ While some studies used a 2-tier comparison (normal vs abnormal BMD), others used a 3-tier comparison (normal vs osteopenia vs osteoporosis). In the current study after mean follow up of 8 years, 12 (52%) had normal bone density, 5 (22%) had osteopenia and 6 (26%) had osteoporosis. While a 3-tier comparison can provide a more detailed and comprehensive assessment of bone health following OLT, we preferred to use the 2-tier comparison because of the relatively small number of patients.

The variation in the prevalence of abnormal BMD between different studies may be attributed to:

1- Different periods of exposure to steroids: patients with reduced bone mass are more likely to have been treated for acute rejection and to have had greater cumulative exposure to prednisone. Patients who are either corticosteroid-dependent or receiving repetitive course of steroids are at greatest risk for reduced bone mass. In the current study, 55% of patients with low BMD were on steroids at the time of DEXA scan compared to 8% of patients with normal BMD; however, steroids was only significant in the univariate analysis but not on multivariate analysis. This highlights the potential effect of steroids on BMD following OLT.
2- Different definitions of BMD: The lack of standardized definition of low BMD where different studies utilized different cutoffs for z-score.
3- Study design: most studies lacked longitudinal follow up, similar to the current study. This may be attributed to the fact that DEXA scan has not been uniformly recommended as part of routine work up for OLT patients.
4- While longitudinal follow up of BMD is missing, we observed improvement in the mean height z-score over time reaching a peak at 10 years after OLT. BMD may have exhibited a similar pattern. However, a prior report failed to demonstrate a correlation between height and BMD.^[33]^
5- Since most studies were retrospective, they are liable to significant selection bias where healthier patients who survived longer and had longer follow up may skew the results.
6- The difference in etiology of end stage liver disease where patients with cholestatic liver disease tend to have worse bone function and therefore may have lower BMD after OLT.

The effect of steroids on BMD in children after OLT remains controversial.^[25,34,35]^ Some studies showed that the use of steroids markedly influenced BMD if administered for >1 to 2 years in doses >0.2 mg/kg/d.^[25,36]^ Other studies showed minimal or no effect of steroids on bone health.^[26,37]^ Few studies utilized steroid-free protocols, and therefore, it is hard to draw any conclusions.^[38]^ Patients with reduced bone mass were more likely to have been treated for acute rejection, and to have had greater cumulative exposure to steroids during the year preceding the measurement of their BMD.^[25]^

The persistence of abnormal BMD in more than half of the patients raises the question whether those patients develop renal tubular acidosis^[11]^ with subsequent bone resorption. However, the laboratory assessment in the current study did not show evidence suggestive of acid base disorder or significant differences between patients with normal BMD and those with abnormal BMD.

We found that on univariate analysis, weight SDS at last follow up, Hb at last follow up, platelets at last follow up were associated with better BMD while being on steroids at last follow up was associated with poor BMD. Nevertheless, none of these variables was significant on multivariable analysis, likely due to small sample size. Higher weight, Hb level and platelet count may reflect better overall health and nutritional status and therefore may be associated with better BMD.

In agreement with our findings, low weight has been associated with BMD since these patients have smaller bones.^[39]^ Association between BMD and short stature have also been previously described. In contrast, Noble et al did not demonstrate a significant association between height, weight, or BMI z-scores with BMD. In contrast, another study showed that low BMI following transplant was associated with poor bone health.^[26]^

Age at transplant was not associated with BMD in our cohort, similar to previous reports.^[33]^ However, another study showed that children who underwent OLT who are 10 years or older at the time of transplant may have the greatest risk of poor bone health in the future.^[26,33]^ Whether time since transplant predicts BMD or not remains controversial.^[4,25,26,33]^

In children, vitamin D levels are low both pre- and post OLT but improve during the first year.^[30,40]^ In the current study, the median vitamin D level was low among patients with normal BMD and those with abnormal BMD. Additionally, approximately 86% of patients were vitamin D-deficient regardless of their BMD status as determined by DEXA scan. This is in contrast to prior studies, where Guthery et al showed only 6% of patients with low vitamin D level and only one of these had low BMD in their study of 87 patients. The current study, however, is limited by the lack of appropriate data about the nutritional status of patients before and after transplantation, as well as exposure to sunlight. Additionally, no information was available about whether the patients were receiving vitamin D supplementation on regular basis or not.

This study has several limitations. The small number of patients who underwent DEXA scans is a major limitation. However, we found that approximately half of the patients who underwent DEXA scans had abnormal BMD, a finding that can lead incorporation of DEXA scans into the routine surveillance protocols for patients after OLT. Second, the lack of longitudinal follow up for evaluation of bone health. Retrospective fracture history is open to recall bias since very low number of children experienced fractures compared to other studies. Our study can be susceptible to selection bias since we enrolled only patients who presented to their annual follow up, we selected those who are alive and out of hospital. It is possible that patients who were not included, those who were hospitalized or non-survivors, had reduced bone mass, and, therefore, this study may have underestimated the prevalence of reduced bone mass among young transplant recipients. On the other hand, the mean time since transplantation for this sample is 8 years. Thus, this sample is likely a good estimate for the prevalence of reduced bone mass in long-term survivors after liver transplantation (LT). Lack of controls may also confound our results. Additionally, this is a single center experience which limits the generalizability of results.

In conclusion, 48% of patients who underwent OLT demonstrated poor bone health as measured by DEXA scans. This highlights the importance of assessment of bone health as part of routine work up for patients before and after OLT.

## Author contributions

**Conceptualization:** Magd A. Kotb, Lubna A. Fawaz, Hesham Abd El Kader, Gamal El Tagy, Alaa F. Hamza.

**Data curation:** Magd A. Kotb, Rania A. Zeitoun, Yomna M. Shaalan, Nazira Aly, Hesham Abd El Kader, Gamal El Tagy, Haytham Esmat, Alaa F. Hamza, Hend Abd El Baky.

**Formal analysis:** Magd A. Kotb, Lubna A. Fawaz, Rania A. Zeitoun, Yomna M. Shaalan, Nazira Aly, Gamal El Tagy, Haytham Esmat, Hend Abd El Baky.

**Funding acquisition:** Magd A. Kotb.

**Investigation:** Magd A. Kotb, Rania A. Zeitoun, Yomna M. Shaalan, Nazira Aly, Hesham Abd El Kader, Haytham Esmat, Alaa F. Hamza, Hend Abd El Baky.

**Methodology:** Magd A. Kotb, Lubna A. Fawaz, Rania A. Zeitoun, Yomna M. Shaalan, Nazira Aly, Hesham Abd El Kader, Gamal El Tagy, Haytham Esmat, Alaa F. Hamza, Hend Abd El Baky.

**Project administration:** Magd A. Kotb, Lubna A. Fawaz, Yomna M. Shaalan, Hend Abd El Baky.

**Resources:** Rania A. Zeitoun, Yomna M. Shaalan, Nazira Aly, Hesham Abd El Kader, Gamal El Tagy, Alaa F. Hamza, Hend Abd El Baky.

**Software:** Rania A. Zeitoun, Nazira Aly, Hend Abd El Baky.

**Supervision:** Magd A. Kotb, Lubna A. Fawaz, Yomna M. Shaalan, Hesham Abd El Kader, Gamal El Tagy, Haytham Esmat, Alaa F. Hamza.

**Validation:** Lubna A. Fawaz, Rania A. Zeitoun, Yomna M. Shaalan, Gamal El Tagy, Haytham Esmat, Hend Abd El Baky.

**Visualization:** Magd A. Kotb, Lubna A. Fawaz, Hesham Abd El Kader, Alaa F. Hamza.

**Writing – original draft:** Magd A. Kotb, Lubna A. Fawaz, Yomna M. Shaalan, Hesham Abd El Kader, Hend Abd El Baky.

**Writing – review & editing:** Magd A. Kotb, Lubna A. Fawaz, Rania A. Zeitoun, Yomna M. Shaalan, Nazira Aly, Hesham Abd El Kader, Gamal El Tagy, Haytham Esmat, Alaa F. Hamza, Hend Abd El Baky.

## Supplementary Material


