# Supplementary material for: Bone demineralization in a cohort of Egyptian pediatric liver transplant recipients: Single center pilot study
Source: Medicine (Baltimore). 2022 Nov 11;101(45):e31156. doi: 10.1097/MD.0000000000031156 (PMC10662835; doi:10.1097/MD.0000000000031156)
Supplement: Supplementary file 1 [file medi-101-e31156-s001.pdf]

Supplementary Table 1: Immunosuppression medications received by patients who presented to the annual follow up

**Number of Immunosuppression agents**	**N (%)**
Single	5 (21.7)
Multiple	17 (73.9)
None	1 (4.3)
**Type of immunosuppression**	**n (%)**
Steroids	7 (29.2)
Imuran	7 (29.2)
Prograph	21(87.5)
Cellcept	11 (45.8)
Everolimus	1 (4.2)
