# Supplementary material for: Bone demineralization in a cohort of Egyptian pediatric liver transplant recipients: Single center pilot study
Source: Medicine (Baltimore). 2022 Nov 11;101(45):e31156. doi: 10.1097/MD.0000000000031156 (PMC10662835; doi:10.1097/MD.0000000000031156)
Supplement: Supplementary file 2 [file medi-101-e31156-s002.pdf]

**Supplementary Table 2:** Laboratory assessment of bone health of patients who presented for the annual follow up	
Vitamin D level, mean (SD)	12.79 (±10.63)
Vitamin D status, n (%)	
Normal	2 (9.5%)
Insufficient	1 (4.8%)
Deficient	18 (85.7%)
PTH in folds, mean (SD)	1.52 (±1.11)
Calcium, mean (SD)	9.04 (±0.84)
Ionized calcium, mean (SD)	1.5 (± 0.9)
Serum Phosphorus, mean (SD)	4.22 (±0.75)
Serum Mg, mean (SD)	1.82 (2.89)
Serum Cl, mean (SD)	103.32 (±4.22)
Anion Gap, mean (SD)	9.6 (±4.22)
Serum Alkaline phosphatase, mean (SD)	1.29 (±0.79)
