# Supplementary material for: Bone demineralization in a cohort of Egyptian pediatric liver transplant recipients: Single center pilot study
Source: Medicine (Baltimore). 2022 Nov 11;101(45):e31156. doi: 10.1097/MD.0000000000031156 (PMC10662835; doi:10.1097/MD.0000000000031156)
Supplement: Supplementary file 3 [file medi-101-e31156-s003.pdf]

DEXA scan	Mean (SD)
L1 BMD, mean (SD) (g/cm2)	0.76 (±0.28)
L2 BMD, mean (SD) (g/cm2)	0.77 (±0.29)
L3 BMD, mean (SD) (g/cm2)	0.76 (±0.28)
L4 BMD, mean (SD) (g/cm2)	0.73 (±0.25)
L1- L2, mean (SD) (g/cm2)	0.75 (±0.22)
L1-L3, mean (SD) (g/cm2)	0.76 (±0.23)
L1-L4, mean (SD) (g/cm2)	0.73 (±0.23)
L2-L3, mean (SD) (g/cm2)	0.76 (±0.24)
L2-L4, mean (SD) (g/cm2)	0.76 (±0.24)
L3-L4, mean (SD) (g/cm2)	0.72 (±0.2)
Total Lumbar BMD	0.75 (±0.26)
Lumbar Z-score	-1.3(±1.5)
Normal	12 (±52.2%)
Osteopenia	5 (±21.7%)
Osteoporosis	6 (±26.1%)
