# Supplementary material for: Bone demineralization in a cohort of Egyptian pediatric liver transplant recipients: Single center pilot study
Source: Medicine (Baltimore). 2022 Nov 11;101(45):e31156. doi: 10.1097/MD.0000000000031156 (PMC10662835; doi:10.1097/MD.0000000000031156)
Supplement: Supplementary file 4 [file medi-101-e31156-s004.pdf]

**DEXA scan**	**Normal DEXA scan**	**Abnormal DEXA scan**	**P-value**
L1 BMD, median(range)(g/cm2)	0.77 (0.6-1.52)	0.5 (0.4-0.9)	0.005
L2 BMD, median(range)(g/cm2)	0.82 (0.6-1.4)	0.59 (0.4-1.02)	0.006
L3 BMD, median(range) (g/cm2)	0.84 (0.58-0.13)	0.54 (0.4-1.08)	0.006
L4 BMD, median(range) (g/cm2)	0.81 (0.51-1.23)	0.55(0.38-0.93)	0.03
L1- L2, median(range) (g/cm2)	0.74 (0.6-1.23)	0.66(0.46-0.98)	0.30
L1-L3, median(range) (g/cm2)	0.75 (0.59-1.23)	0.67 (0.44-1.02)	0.24
L1-L4, median(range) (g/cm2)	0.72 (0.58-1.23)	0.68 (0.41-0.99)	0.13
L2-L3, median(range) (g/cm2)	0.74 (0.59-1.27)	0.68 (0.42-1.05)	0.24
L2-L4, median(range) (g/cm2)	0.73 (0.57-1.25)	0.69 (0.42-1.01)	0.17
L3-L4, median(range) (g/cm2)	0.71 (0.54-1.15)	0.7 (0.41-1)	0.42
Total Lumbar BMD	0.83 (0.58-1.32)	0.57 (0.41-1.01)	0.006
Lumbar Z-score	-0.45 (-0.8-1.1)	-2.3(-4.8- -1.1)	0.0001
