# Supplementary material for: Bone demineralization in a cohort of Egyptian pediatric liver transplant recipients: Single center pilot study
Source: Medicine (Baltimore). 2022 Nov 11;101(45):e31156. doi: 10.1097/MD.0000000000031156 (PMC10662835; doi:10.1097/MD.0000000000031156)
Supplement: Supplementary file 5 [file medi-101-e31156-s005.pdf]

Supplementary Table 5: Laboratory assessment of bone health (patients with normal DEXA scan versus abnormal)

**Bone Health (last follow up)**	**Normal DEXA scan**	**Abnormal DEXA scan**	**p-value**
History of fractures, n (%)	1 (8.3)	2 (18.2)	0.59
Fracture site			0.20
Forearm, n (%)	0	2 (100)	
Lower Limb, n (%)	1 (100)	0	
Vitamin D level	10.9 (4.9-14.6)	9.7 (3-47.8)	0.93
Vitamin D status			0.58
Normal	1 (10)	1 (10)	
Insufficient	0	1 (10)	
Deficient	9 (90)	9 (80)	
PTH in folds, median(range)	1.09 (1-2.5)	1.1 (1-5.9)	0.73
Calcium, median(range)	9 (7.8-11.2)	9.3 (6.5-9.6)	0.33
Ionized calcium, median(range)	1.1 (1.1-4.2)	1.2(1.1-1.2)	0.58
Serum Phosphorus, median(range)	4.1 (2.6-5.2)	4.3 (2.8-5.5)	0.30
Serum Mg, median(range)	1.95 (1-2.3)	1.9 (1.5-2.3)	0.45
Serum Cl, median(range)	102 (100-106)	104 (101-110)	0.03
Anion Gap, median(range)	8.7 (6.5-15.8)	9.9 (0.6-13.9)	0.83
Serum Alkaline phosphatase in folds, median(range)	1 (1-3.6)	1 (1-3.9)	0.08
