# Supplementary material for: Bone demineralization in a cohort of Egyptian pediatric liver transplant recipients: Single center pilot study
Source: Medicine (Baltimore). 2022 Nov 11;101(45):e31156. doi: 10.1097/MD.0000000000031156 (PMC10662835; doi:10.1097/MD.0000000000031156)
Supplement: Supplementary file 6 [file medi-101-e31156-s006.pdf]

Supplementary Table 6: Univariable logistic regression analysis modeling variables associated with abnormal DEXA scan

	**Odds Ratio (OR)**	**95% Confidence Interval (CI)**	**p-value**
**Pre-operative variables**			
Sex	5.25	0.87-31.5	0.07
Age at transplantation	0.69	0.21-2.23	0.54
Pre-operative weight	0.35	0.12- 1.02	0.05
Pre-operative height	0.78	0.44-1.38	0.39
Pre-operative BMI	0.46	0.2-1.06	0.07
Pre-operative albumin	0.51	0.18-1.4	0.20
Pre-operative ALT (folds)	1.6	0.46-5.55	0.45
Pre-operative T. Bilirubin	0.99	0.91-1.07	0.84
Pre-operative D. bilirubin	1.02	0.89-1.16	0.76
Pre-operative creatinine	0.93	0.54-1.62	0.82
Pre-operative PT	1.02	0.86-1.22	0.75
Pre-operative INR	1.2	0.25- 5.81	0.81
PELD	1.05	0.94- 1.16	0.34
Diagnosis	0.96	0.82- 1.13	0.68
Donor relation	0.58	0.09-3.5	0.55
Donor Gender	0.28	0.05-1.59	0.15
**Operative variables**			
Duration in ICU	1.02	0.9-1.15	0.71
Duration in room	1	0.91- 1.1	0.91
Complications	2.25	0.32-15.7	0.41
Acute rejection	2.85	0.4-20.1	0.29
Chronic rejection	2.4	0.44-12.9	0.3
Biliary complications	4.12	0.35-47.3	0.25
Vascular complications	1.87	0.24-14	0.54
**Immunosuppression**			

Steroids received	9.16	0.86-97.6	0.06
**Last follow up**			
Height SDS	0.76	0.44- 1.33	0.35
Weight SDS	0.45	0.2-0.99	0.04
BMI SDS	0.45	0.19- 1.05	0.06
AST (folds)	0.93	0.58- 1.49	0.78
ALT (folds)	0.92	0.54-1.5	0.78
GGT (folds)	0.94	0.7-1.2	0.73
T. Bilirubin	0.9	0.65- 1.2	0.53
D. Bilirubin	0.85	0.52-1.37	0.50
Glucose	0.97	0.89- 1.05	0.54
Urea	0.96	0.88- 1.04	0.39
Creatinine	1.5	0.11- 19.2	0.75
Na	0.93	0.76- 1.15	0.55
K	4.66	0.38- 56.82	0.22
HCO3	0.97	0.64- 1.46	0.90
Mg	0.67	0.03- 13.67	0.80
Anion gap	0.93	0.67- 1.28	0.67
CL	1.6	0.97- 2.62	0.06
PT	1.21	0.71- 2.06	0.46
INR	1.08	0.002- 420.52	0.97
Albumin	0.33	0.03- 3.32	0.34
Hb	0.33	0.12- 0.89	0.03
Hct	0.89	0.71- 1.11	0.31
Platelets	0.98	0.96- 0.99	0.02
WBCs	0.88	0.6- 1.27	0.50
Creatinine clearance	0.98	0.95- 1.01	0.21
Single immunosuppression	0.2	0.49- 3.16	0.18
Multiple immunosuppression	4.99	0.46- 54	0.18

Steroids	13.2	1.23-140.67	0.03
Fractures	2.44	0.18- 31.5	0.49
Ca	0.74	0.26- 2.04	0.56
P	1.71	0.52- 5.61	0.37
ALP (folds)	1.24	0.42- 3.66	0.69
PTH (folds)	1.49	0.54- 4.06	0.43
Vitamin D	1.05	0.94- 1.18	0.35
Vitamin D status	0.44	0.18-22.05	0.53
