# Supplementary material for: Bone demineralization in a cohort of Egyptian pediatric liver transplant recipients: Single center pilot study
Source: Medicine (Baltimore). 2022 Nov 11;101(45):e31156. doi: 10.1097/MD.0000000000031156 (PMC10662835; doi:10.1097/MD.0000000000031156)
Supplement: Supplementary file 7 [file medi-101-e31156-s007.pdf]

**Supplementary Table 7: Multivariable** logistic regression analysis modeling variables associated with abnormal DEXA scan

| **Variable** | **Odd Ratio (OR)** | **95% Confidence Interval (CI)** | **p- value** |
|---|---|---|---|
| Pre-operative weight SDS | 0.11 | 0.003- 3.2 | 0.20 |
| Weight SDS at last follow up | 1.38 | 0.36- 5.21 | 0.63 |
| Hb at last follow up | 0.18 | 0.01- 1.79 | 0.14 |
| Platelets at last follow up | 0.95 | 0.9- 1 | 0.09 |
| Receiving steroids at last follow up | 161.68 | 0.04- 58 | 0.22 |
